# Supplementary material for: Ultrashort vertical-channel MoS2 transistor using a self-aligned contact
Source: Nat Commun. 2024 Jan 2;15:165. doi: 10.1038/s41467-023-44519-x (PMC10761794; doi:10.1038/s41467-023-44519-x)
Supplement: Supplementary file 1 — Supplementary Information [file 41467_2023_44519_MOESM1_ESM.pdf]

Supplementary Information for

Ultrashort vertical-channel MoS_2 transistor using a self-aligned contact

Liting Liu[1], Yang Chen[1], Long Chen[1], Biao Xie[1], Guoli Li[1,*], Lingan Kong[1], Quanyang Tao[1], Zhiwei Li[1], Xiaokun Yang[1], Zheyi Lu[1], Likuan Ma[1], Donglin Lu[1], Xiangdong Yang[2], Yuan Liu[1,*]

[1]Key Laboratory for Micro-Nano Optoelectronic Devices of Ministry of Education, School of Physics and Electronics, Hunan University, Changsha 410082, China.

[2]Institute of Micro/Nano Materials and Devices, Ningbo University of Technology, Ningbo, 315211, China.

[*]e-mail: yuanliuhnu@hnu.edu.cn; liguoli_lily@hnu.edu.cn

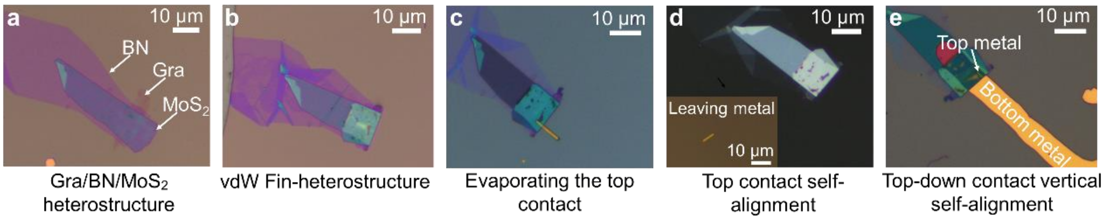

Supplementary Fig. 1. The optical images of the fabrication process. a, Stacking of MoS_2/BN/graphene heterostructure. **b**, Formation of vdW Fin-heterostructure. **c**, Deposition of the top metal contact. **d**, Peeling off the folded heterostructure. **e**, Transfer of the folded heterostructure to realize self-aligned vertical contact.

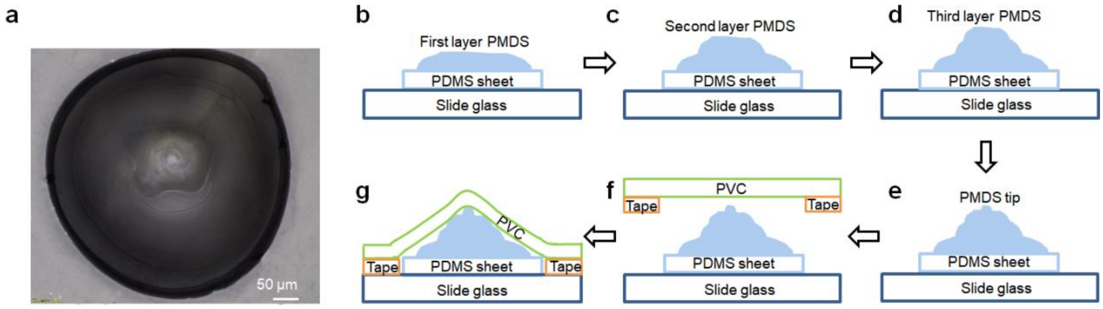

Supplementary Fig. 2. The characterization and fabrication processes of PDMS tip. a, The image of the bumped PDMS stamp which used to fold the heterostructure. **b–g**, The fabrication process of the PDMS tip, comprising six essential steps: (**b**) dropping first layer PDMS liquid using tungsten needle with diameter of 2 mm, (**c**) dropping the second layer PDMS liquid using the tungsten needle with diameter of 600 μm, (**d**) dropping the third layer PDMS liquid using the tungsten needle with diameter of 50 μm, (**e**) dropping the fourth layer PDMS liquid using the tungsten needle with diameter of 400 nm, (**f**, **g**) coating the PVC layer on top of PDMS tip to enhance the adhesion force.

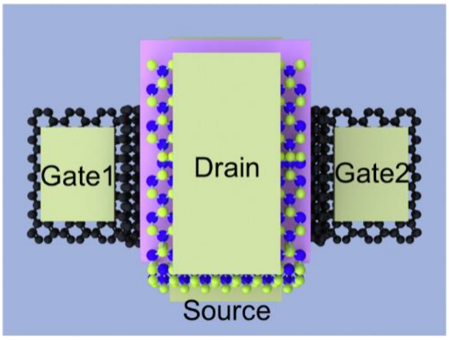

Supplementary Fig. 3. The top view schematic of the self-alignment device. During the folding process, the actual graphene gate extends to both sides of the folded heterostructure, forming two tails outside BN for gate contact (Gate 1 and Gate 2).

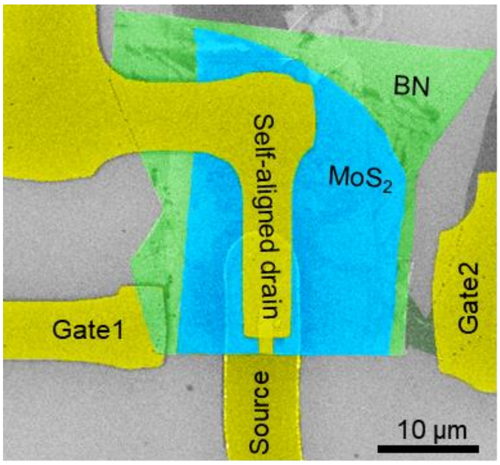

Supplementary Fig. 4. False-colored SEM image of the device. Blue color represents MoS_2, green color represents BN and yellow color represents the electrodes.

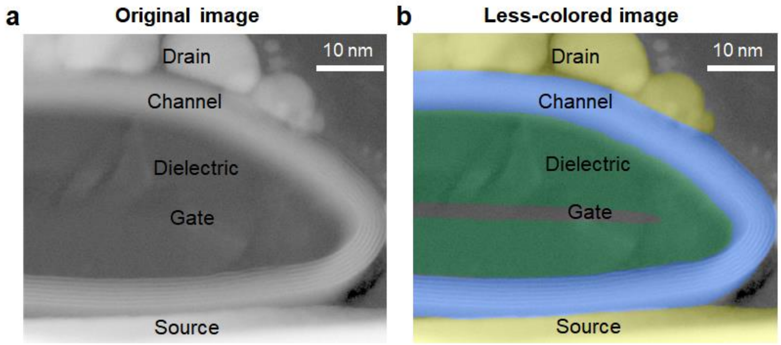

Supplementary Fig. 5. TEM image of self-aligned device. a, Original TEM image of the self-aligned device. **b**, Corresponding TEM image with less false-coloring.

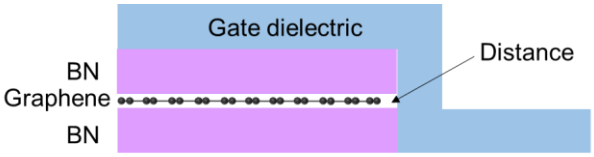

Supplementary Fig. 6. Graphene side gate structure (BN/graphene/BN) through conventional layer-by-layer stacking processes. Within this process, it is hard to precisely align the graphene with the edge of BN, resulting in large distance between the graphene gate and the BN edge.

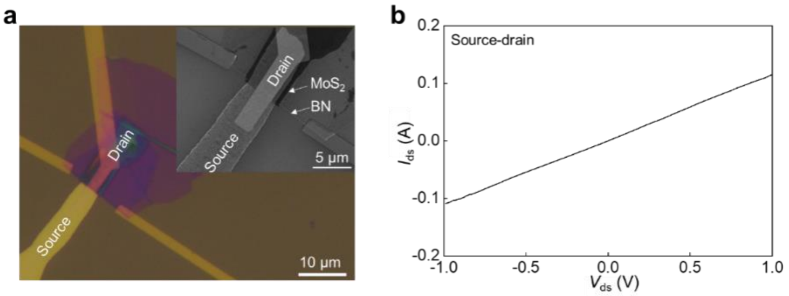

Supplementary Fig. 7. Characterization of the tilted angle deposition device. a, The optical image and corresponding SEM image of the folded heterostructure device using tilted angle deposition for source-drain contact. **b**, The electrical characteristic of corresponding device, showing the short circuit between the source and drain.

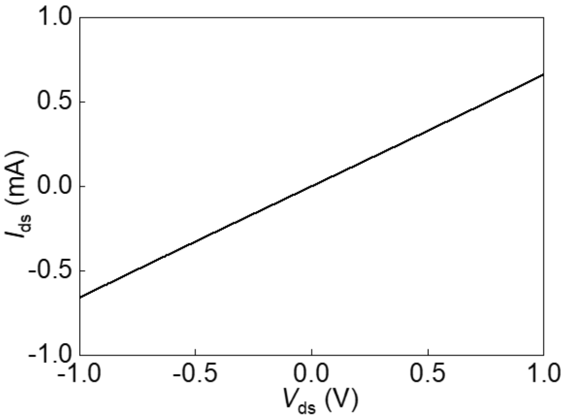

Supplementary Fig. 8. The conductivity of graphene gate. The two terminal device exhibits low resistance, demonstrating enough conductivity as a gate electrode.

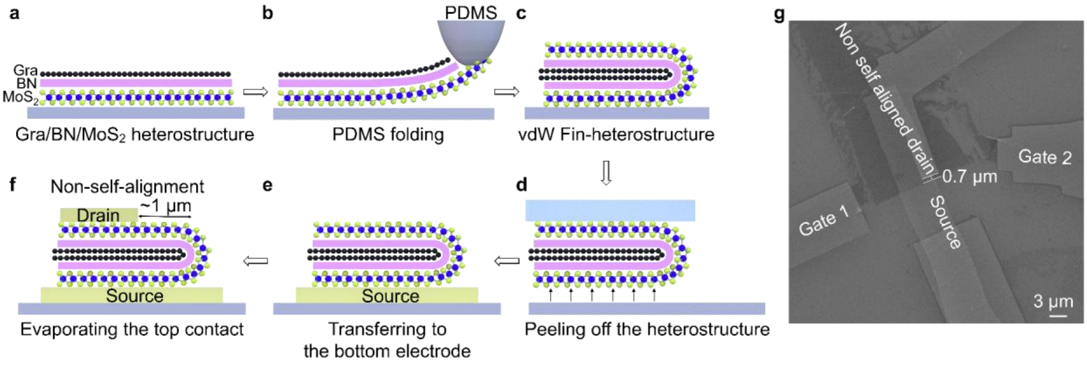

Supplementary Fig. 9. Fabrication processes and characterization of the non-self-aligned device. a–f, Fabrication processes of the control device without self-alignment. Including six steps: stacking of MoS_2/BN/graphene heterostructure (**a**), PDMS folding (**b**), creation of vdW fin-heterostructure (**c**), peeling-off the vdW fin-heterostructure (**d**), transferring the folded heterostructure to the bottom metal (**e**), and defining the top contact by electron beam lithography (**f**). **g**, The SEM image of the non-self-aligned device.

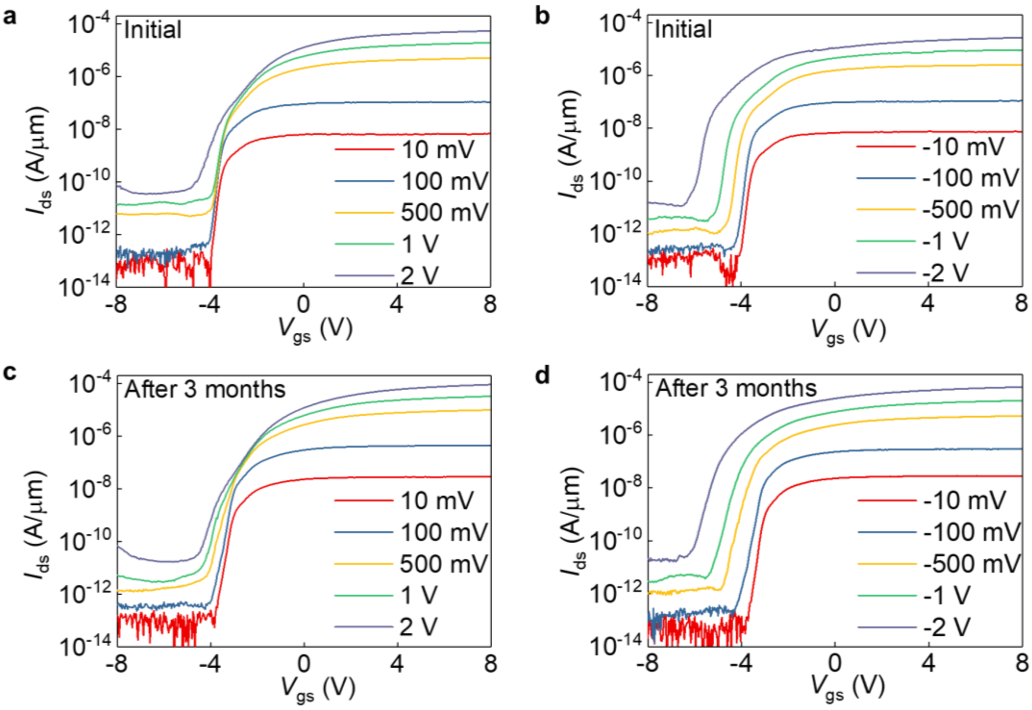

Supplementary Fig. 10. Stability measurement of a typical self-aligned contact device. a, **b**, Transfer characteristics of an initial self-aligned contact device in both positive (**a**) and negative (**b**) bias regions. **c**, **d**, Transfer characteristics of a self-aligned contact device which storing at ambient condition for three months.

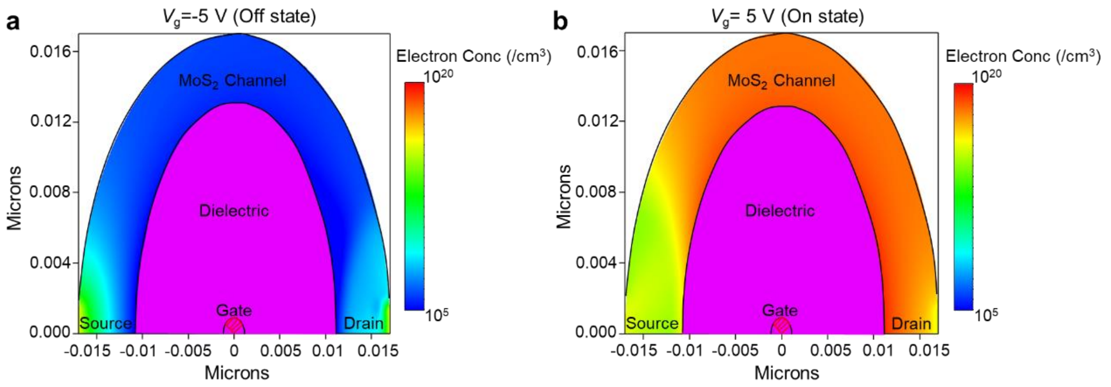

Supplementary Fig. 11. The simulated electron density distribution of vertical device.

a, The electron density distribution of the MoS_2 channel at off-state. **b**, The electron density distribution of the MoS_2 channel at on-state.

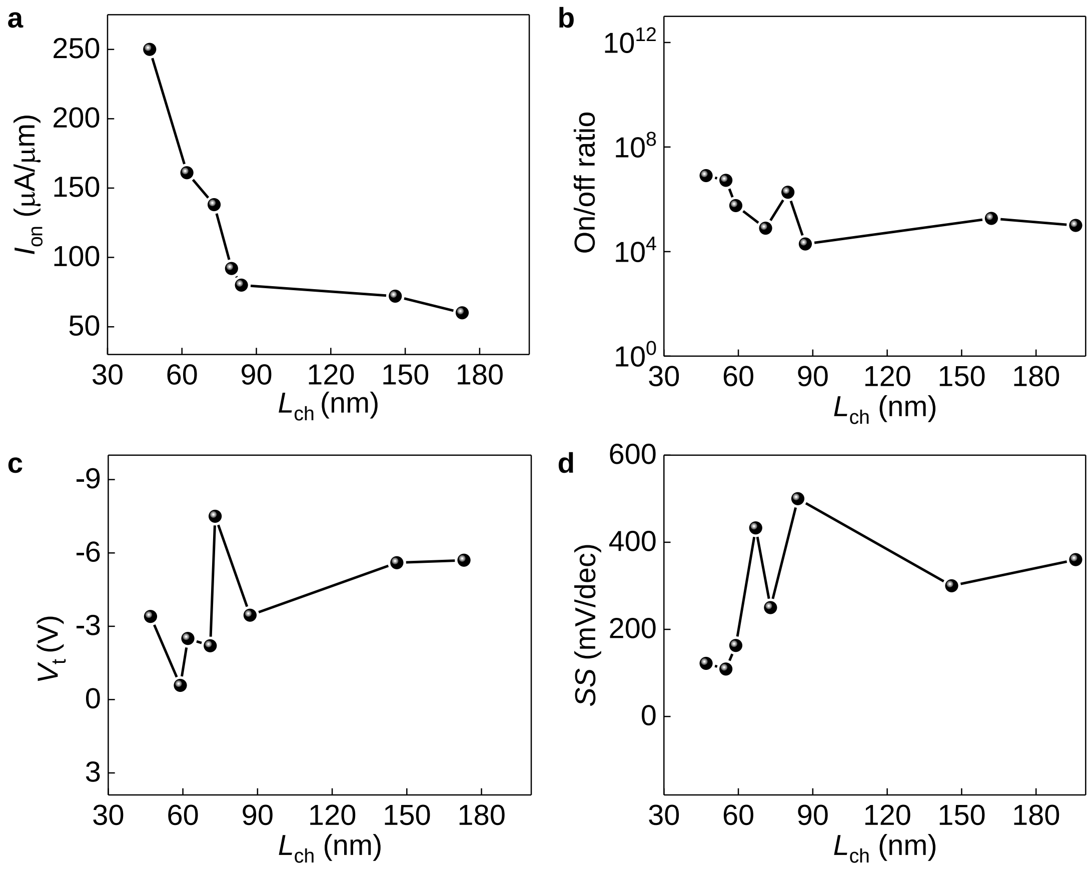

Supplementary Fig. 12. The electrical performance of self-aligned devices as a function of channel length. a, The on-state current density of the self-aligned devices with the relationship of channel length. **b**, The on-off ratio of the self-aligned devices with the relationship of channel length. **c**, The threshold voltage of the self-aligned devices with the relationship of channel length. **d**, The subthreshold swing of the self-aligned devices with the relationship of channel length. The channel length (L_{ch}) here is extracted by calculating the distance between edges of source and drain along the curvature.

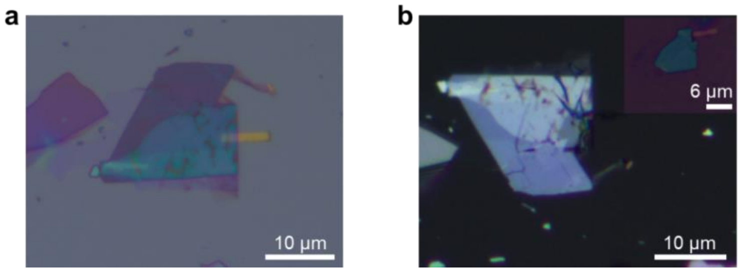

Supplementary Fig.13. Self-aligned process for thinner heterostructure. a, Optical image of the thinner heterostructure (~25 nm thick after folding) with top contact deposited. **b**, Optical image of the structure peeling from substrate, where part of the flake is left at the original substrate owing to the similar thickness of the heterostructure and the top contact metal.

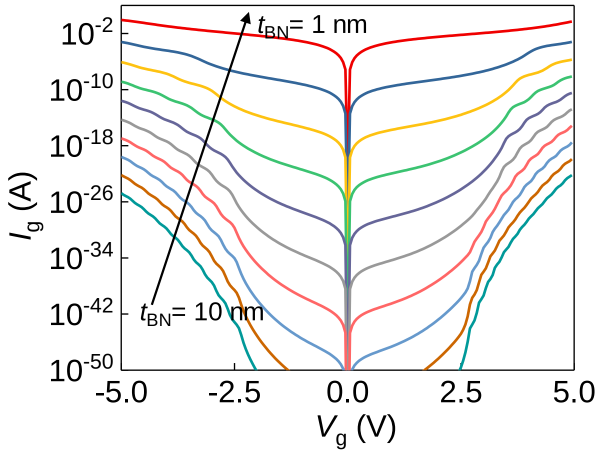

Supplementary Fig.14. The simulation results of gate leakage currents with different BN thickness from 1 nm to 10 nm.

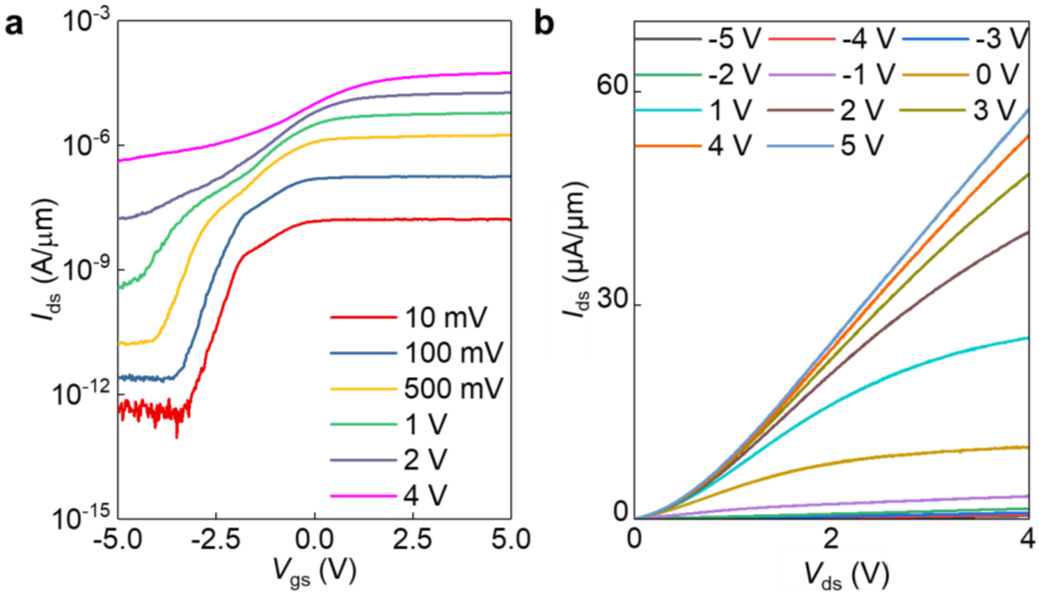

Supplementary Fig. 15. Self-aligned device operated on the SiO_2. a, I_{ds}–V_{gs} transfer characteristics of a self-aligned device on SiO_2. **b**, I_{ds}–V_{ds} output characteristics of a self-aligned device on SiO_2. The thickness of MoS_2 is 14 layers, channel length is around 80 nm.
